# Comparing lateral flow testing with a rapid RT‐PCR method for SARS‐CoV‐2 detection in the United Kingdom—A retrospective diagnostic accuracy study

**DOI:** 10.1002/hsr2.811

**Published:** 2022-09-14

**Authors:** Andrew Taylor, Ronan Calvez, Mark Atkins, Colin G. Fink

**Affiliations:** ^1^ Micropathology Ltd., Venture Centre University of Warwick Science Park Coventry UK; ^2^ University of Warwick Coventry West Midlands UK

**Keywords:** diagnostics, lateral flow, RT‐PCR, SARS‐CoV‐2, sensitivity

## Abstract

**Background and Aims:**

In late 2019, severe acute respiratory syndrome coronavirus 2 (SARS‐CoV‐2) emerged in Wuhan, China. Rapid global spread led to the coronavirus disease 2019 (Covid‐19) pandemic. Accurate detection of SARS‐CoV‐2 has become a vitally important tool in controlling the spread of the virus. Lateral flow devices (LFDs) offer the potential advantage of speed and on‐site testing. The sensitivity of these devices compared to reverse transcription‐polymerase chain reaction (RT‐PCR) has been questioned.

**Methods:**

We compared the sensitivity of the Innova LFD, used widely in the United Kingdom, with our rapid RT‐PCR method using stored positive samples. Samples with a range of viral loads (original Ct values 18.9–36.5) were tested.

**Results:**

The Innova LFD was found to be 6000–10,000 times less sensitive than RT‐PCR for SARS‐CoV‐2 detection. Overall, the LFD detected 46.2% of the positives detected by RT‐PCR, with 50% of these observed to be weak LFD positives. At lower viral loads, such as 10,000–100,000 RNA copies/ml, the LFD detected 22.2% of positives. In addition, two strong positives (3 and 1.5 million RNA copies/ml) were not detected by the LFD.

**Conclusion:**

The argument for use of LFD kits is that they detect infectious virus and hence contagious individuals. However, there is a lack of conclusive evidence supporting this claim. The Innova LFD has been subject to a Class I recall by the US Food and Drug Administration, but is still approved and widely used in the United Kingdom.

## 1 INTRODUCTION

In late 2019, a novel coronavirus emerged in Wuhan, China. This virus was designated severe acute respiratory syndrome coronavirus‐2 (SARS‐CoV‐2) and rapid global spread led to the coronavirus disease 2019 (Covid‐19) pandemic. Symptoms vary between individuals, but the most common symptoms are fever, dry cough, headache, fatigue, anosmia, and diarrhea.^1^, ^2^ The United Kingdom has been badly affected with 17.6 million cases and the second highest death toll in Europe (>157,000).^3^ The devastating nature of this pandemic facilitated the need for high‐throughput and accurate diagnostic methods. RT‐PCR protocols for the detection of SARS‐CoV‐2 RNA were published very early in the pandemic with the first publication detailing probe‐based reverse transcription‐polymerase chain reaction (RT‐PCR) assays targeting E, N, and RdRp genes.^4^ Shortly after this, the US Centers for Disease Control and Prevention (CDC) published three assays targeting the N gene.^5^ Many of these published assays have been developed as commercial kits and are used globally for the detection of SARS‐CoV‐2 RNA. We previously developed a sensitive, rapid, United Kingdom Accreditation Service‐accredited RT‐PCR assay utilizing the CDC N1 assay.^6^ RNA is prepared using a rapid heat treatment method, removing the need for time‐consuming and expensive nucleic acid extraction kits.^6^

RT‐PCR has the advantage of being the most sensitive diagnostic technique available and is regarded as the gold standard for SARS‐CoV‐2 detection. As the pandemic progressed, the possibility of using rapid, point‐of‐care devices such as lateral flow devices (LFDs), was investigated. Such kits target viral proteins and claim to detect live viruses and hence infectious individuals.^7^ They offer the advantage that results can be obtained in as little as 30 min without the need for laboratory processing. However, there have been questions about their sensitivity, particularly when compared to RT‐PCR.^8^, ^9^, ^10^ Due to their availability and convenience for self‐testing, LFDs have become very widely used in the United Kingdom and many other countries.

We retrospectively compared the sensitivity of the UK government‐supplied Innova LFD with RT‐PCR using samples that had previously tested as SARS‐CoV‐2 positive (by RT‐PCR) and were subsequently stored at −20°C. Using digital‐droplet PCR (ddPCR) to calibrate clinical samples, we were able to directly compare PCR (in terms of RNA copies/ml) with the LFD currently used in the United Kingdom.

## 2 METHODS

### 2.1 Patient samples

Initially, 62 stored swab samples comprising 52 known PCR positives (original Ct values 18.9–36.5) and 10 negatives (no visible amplification curve) were selected. Samples were initially grouped into Ct categories of <26, 26–30, 30–34, and 34–37, and equal numbers were selected from each category (groupings were modified after retesting). Samples were previously tested as part of our diagnostic service and had been stored at −20°C for up to 3 months. Original samples were either dry swabs, which had been resuspended in 500 µl of 0.1% Igepal CA‐630 (Sigma‐Aldrich), or swabs in Universal Transport Medium. Positives stored in both buffers were tested to show that neither buffer interferes with the LFD. Following the emergence of the delta variant (the dominant variant at the time of this study), eight additional samples, which were confirmed delta variants, were tested. Clinical details were not available for the patients associated with these samples. No samples were excluded from the study. Samples were thawed, allowed to reach room temperature, and then RT‐PCR and LFD reactions were set up concurrently to ensure that samples were not allowed to degrade at room temperature.

### 2.2 RT‐PCR and digital PCR

Samples were thawed and prepared for RT‐PCR using a rapid heat‐treatment method.^6^ Forty microliters of the sample was heat treated and 10 µl was used for PCR. RT‐PCR was carried out in triplicate. To further explore the comparative sensitivity of RT‐PCR and LFD, a strongly positive sample (sample 29) was quantified using droplet digital PCR (ddPCR) (QX200; Bio‐Rad).^11^ This was carried out using a FAM‐labelled N1 probe for SARS‐CoV‐2 together with Bio‐Rad ddPCR Supermix for probes (no dUTP), following the manufacturer's protocol. PCRs were first set up in a final volume of 20 µl using 5 µl of the nucleic acid template and emulsified in Droplet Generation Oil for Probes (Bio‐Rad). The emulsion was transferred to a 96‐well plate and sealed. PCR conditions involved an initial room temperature step (46°C, 60 min), followed by enzyme activation/template denaturation (95°C, 10 min), PCR (40 cycles: 95°C, 30 s and 60°C, 60 s), and a final stage of enzyme deactivation (98°C, 10 min). The droplets were allowed to stabilize at 4°C for at least 10 min before being read on a QX200 Droplet Reader (Bio‐Rad). The QuantaSoft software (Bio‐Rad) was used to determine the numbers of positive and negative droplets and to calculate the concentration of target SARS‐CoV‐2 RNA. The quantified sample was serially diluted from 2.2 million to 220 RNA copies/ml in 0.1% Igepal. Triplicate PCR was then carried out as described above. The lower limit of detection (LLoD) was determined by Probit regression analysis (MedCalc; MedCalc Software Ltd.) using an RNA extract, which was quantified by ddPCR.

### 2.3 Lateral flow

LFDs used for SARS‐CoV‐2 testing in the United Kingdom (Innova rapid antigen test, manufactured by Xiamen Biotime Biotechnology) were directly compared to RT‐PCR. The instructions in the kit state that two drops (50 µl) of extraction buffer should be applied to an LFD. Frozen patient samples were thawed, allowed to reach room temperature, and then 40 µl of the sample was mixed with 160 µl of LFD extraction buffer. From this, 50 µl was applied to LFDs in triplicate using a pipette. Using this method, the same equivalent volume of patient samples was tested on each LFD compared to each single RT‐PCR reaction. Kits were used following the manufacturer's instructions and results were scored independently by two experienced laboratory staff in standardized light conditions. The LLoD was determined as above using Probit regression analysis. The dilution series of sample 29 (described above) was also tested in triplicate on LFDs.

### 2.4 Statistical analysis

Probit regression (MedCalc) was used to determine the LLoD (with a 95% confidence interval) of both methods. These were calculated using either the Ct values or the viral load (in RNA copies/ml) as measured by ddPCR. The correlation between viral load and LFD result was calculated in Excel by first converting the LFD results into a score of 0–3, reflecting the number of replicates with a positive result. The Pearson correlation value (*r*) was then calculated. Pearson correlations were also used to exclude age and gender as potential confounding factors.

### 2.5 Statements

This study was performed at Micropathology Ltd. (University of Warwick Science Park) in April–June 2021. Patient samples were anonymized and were not considered Human Subjects Research due to the quality improvement and public health intent of the work. This study was reviewed and approved by the Micropathology Ltd. Ethics Committee Review Board composed of Professor Sheila Crispin (MA, VetMB, DVA, DVOphthal, DipEVCO, FRCVS), Professor Christopher Dowson (BSc, PhD), Rt Hon Countess of Mar, Most Rev Dr Gordon Mursell (MA, Hon DD), and William N. H. Taylor (BTech)). No additional consent was necessary. The authors have no conflicts of interest or funding to declare. All data are either included in the manuscript or available on request. All authors have read and approved the final version of the manuscript. Andrew Taylor had full access to all of the data in this study and takes complete responsibility for the integrity of the data and the accuracy of the data analysis.

## 3 RESULTS

### 3.1 Sample set

Approximately equal numbers of samples from male and female patients were tested (Supporting Information: Table S1). The age of patients ranged from 4 to 92 (mean = 39) years and the mean Ct value from the original PCR results was 30. No samples with an original Ct value >37 (<3000 RNA copies/ml) were tested. No correlations were observed between age/gender and RT‐PCR Ct value/LFD score (Supporting Information: Table S1).

### 3.2 Comparing RT‐PCR with LFD

**Figure 1:**
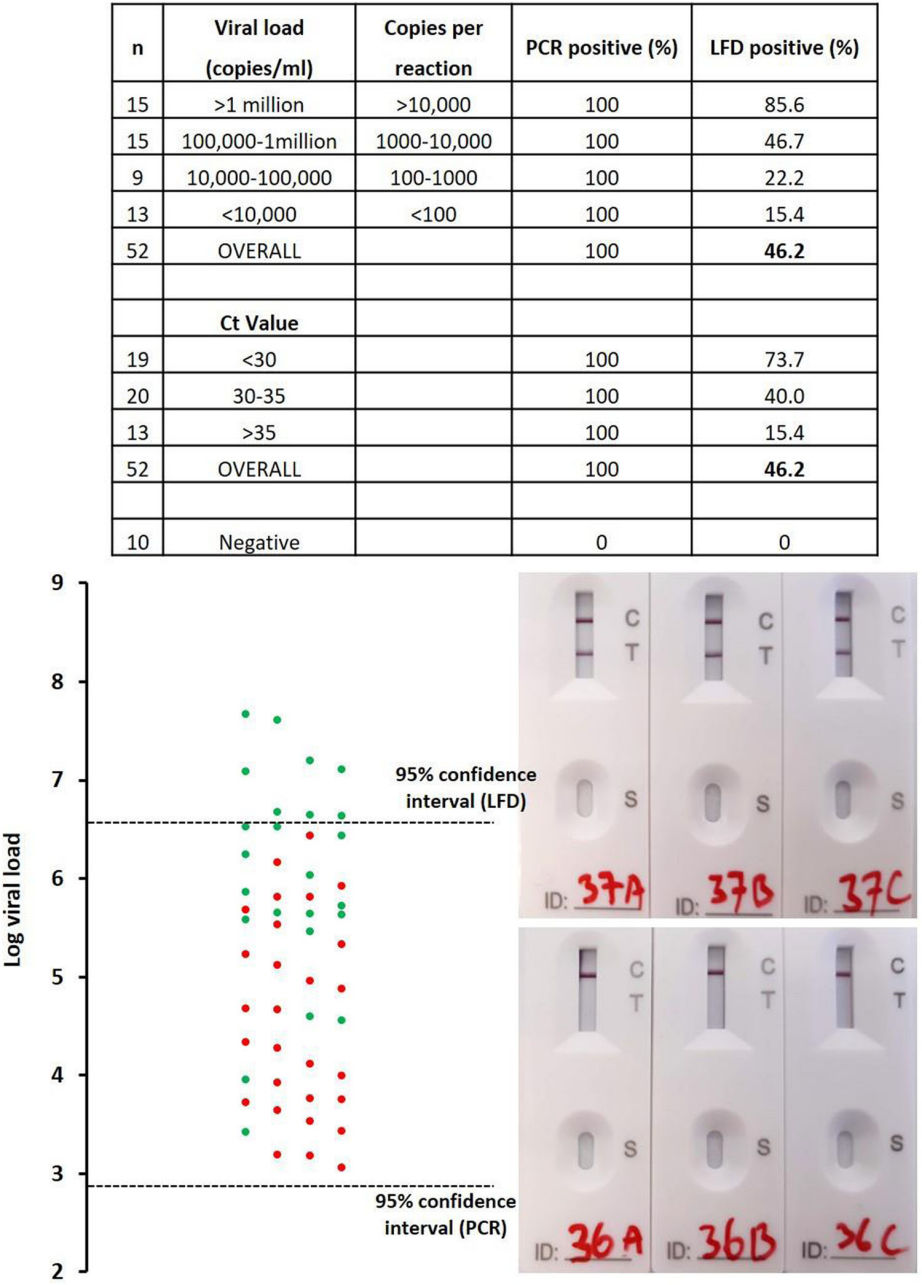
Comparing LFD with RT‐PCR. Samples that were positive in at least two out of the three replicates were classified as positive (green data point). A red data point indicates a sample where 0 or 1 of the three replicates was positive. Of the 52 positive samples tested, 51 were positive in all three RT‐PCR replicates. One sample was positive in two out of three RT‐PCR replicates. Photographs of the LFD device show examples of positive (top) and negative (bottom) test results. The 95% confidence interval was calculated using a Probit regression analysis (Figure 1, 2). LFD, lateral flow device; RT‐PCR, reverse transcription‐polymerase chain reaction.

**Figure 2:**
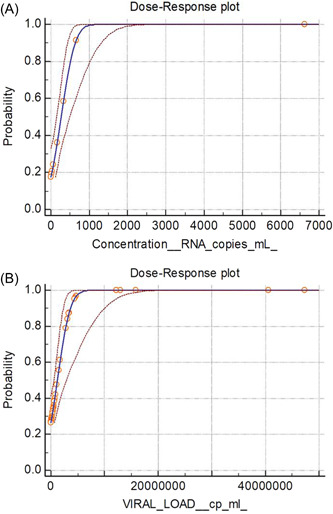
Evaluation of the lower limit of detection of the RT‐PCR assay (A) and the Innova rapid antigen LFD (B) expressed as RNA copies per ml. Probit regression was used to calculate the LLoD of the LFDs using the data summarized in Figure 1, 2 compared to RT‐PCR when multiplexed with an internal control (potato virus Y, PVY). LFD, lateral flow device; LLoD, lower limit of detection; RT‐PCR, reverse transcription‐polymerase chain reaction.

All 10 of the previous PCR negatives were negative on all three replicates of PCR and LFD (Figure 1, 2). Of the 52 previously positive samples, 51 were positive in all three PCR replicates and one sample was positive in two out of three replicates. Of these 52, only 24 (46.2%) returned positive LFD results in at least two out of three replicates. Considering viral load, 85.6% of samples with a viral load of >1 million copies/ml (>10,000 copies per PCR/LFD) were detected by LFD. Surprisingly, two samples in this range, 3 and 1.5 million copies/ml (30,000/15,000 copies per PCR/LFD), were negative in all three LFD replicates. Using an S‐gene dropout PCR (based on the TaqPath kit^12^), it was likely that both of these samples contained the Alpha variant (B1.1.7), the dominant variant at the time of sampling. In the range of 100,000–1 million copies/ml (1000–10,000 copies per PCR/LFD), 46.7% of the samples were positive in at least two of the three replicate LFD tests. The sensitivity of LFD was very limited (22.2%) in the range of 10,000–100,000 RNA copies/ml (100–1000 copies per PCR/LFD) and even lower at <10,000 copies/ml (15.4%). A statistically significant correlation between Ct value (or log viral load) and LFD result was observed (*r* = 0.59, *p* < 0.001).

### 3.3 LOD of LFD and RT‐PCR

Probit analysis (Figure 1, 2) revealed that the LOD of the RT‐PCR assay is 743 RNA copies/ml (95% confidence interval 508–1559 copies/ml; *p* < 0.0001). This equates to 7.4 copies per PCR reaction. The LLoD of the LFD was found to be 4.35 million copies/ml (95% confidence interval 2.75–12.0 million copies/ml; *p* < 0.0001). In terms of RNA copies, this shows that 43,500 copies must be applied to the LFD to ensure a positive result, suggesting that the LFDs were ~6000 times less sensitive than RT‐PCR.

**Figure 3:**
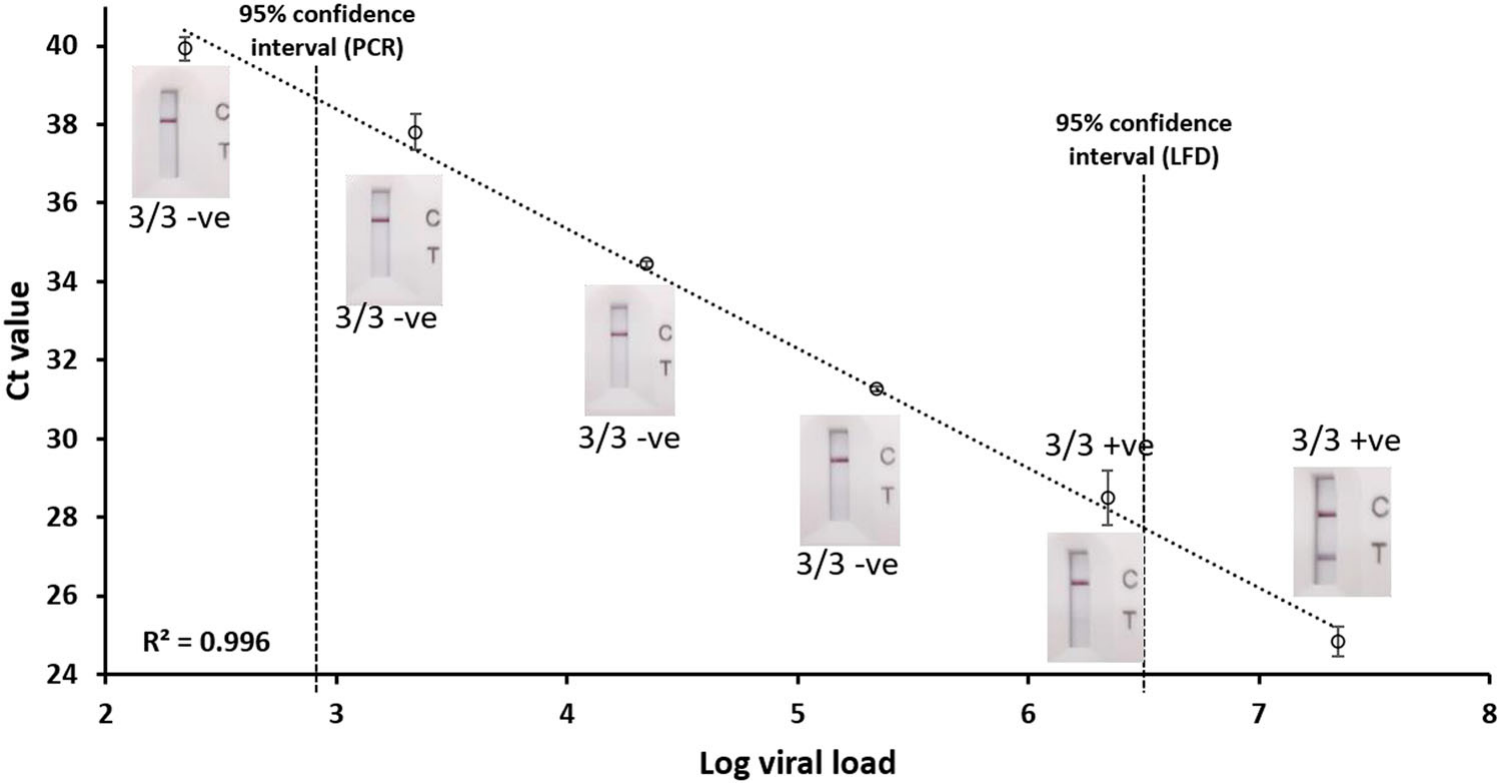
Correlating Ct value (RT‐PCR) with viral load (digital PCR). Results of LFD tests are shown for each dilution. The 95% confidence intervals were calculated based on the Probit analysis (Figure 1, 2). LFD, lateral flow device; RT‐PCR, reverse transcription‐polymerase chain reaction.

To further explore the sensitivity of LFD devices, a dilution series of a strongly positive sample (22 million copies/ml) was tested. A strong correlation between viral load (digital PCR) and Ct value (RT‐PCR) was observed (*r* = −0.998, *p* < 0.001; Figure 3). All LFD tests were negative below 2.2 million copies/ml, although PCR was successful down to 220 copies/ml. This indicates that the PCR test used is at least 10,000 times more sensitive than LFD, which is consistent with the data obtained above.

### 3.4 Detection of the delta variant

Due to the rapid spread of the delta variant in the United Kingdom at the time of the study, eight samples that were confirmed to contain the delta variant were tested by LFD and PCR. The results were comparable with the data described above (Figure 1, 2). Samples with a Ct value of >30 were not detected by LFD (Table 1). Samples with a Ct value of 26–30 were detected but scored as very weak.

**Table 1:** Testing the Innova LFD kit against delta variants of SARS‐CoV‐2

Sample number	Original Ct value	LFD positive (out of 3)	LFD comments	RT‐PCR Ct mean	Ct SEM	Viral load (cp/ml)	Copies per PCR/LFD
63	28.3	3	Very weak	29.6	0.095	765,633	7656
64	25.0	3	Weak	26.5	0.075	7,982,706	79,827
65	21.1	3		22.3	0.095	192,679,428	1,926,794
66	36.8	0		36.7	0.439	3,530	35
67	32.2	0		33.1	0.328	57,215	572
68	26.2	3	Weak	26.5	0.111	7,922,565	79,225
69	18.1	3		18.5	0.027	3,489,133,920	34,891,339
70	19.0	3		21.4	0.171	395,223,450	3,952,234
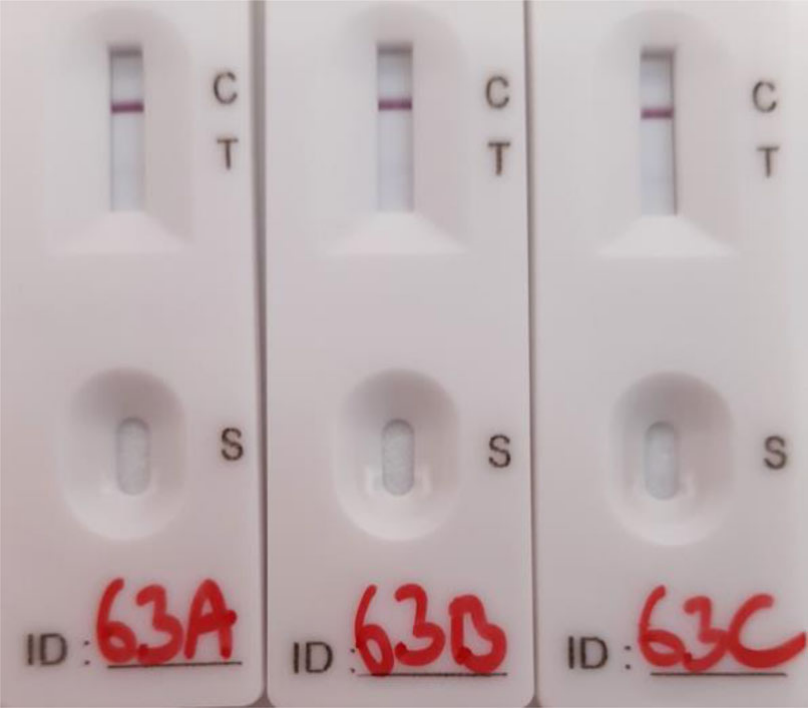							

*Note*: The image illustrates a “weak positive” LFD result. SEM is based on three replicates.

Abbreviations: LFD, lateral flow device; RT‐PCR, reverse transcription‐polymerase chain reaction; SARS‐CoV‐2, severe acute respiratory syndrome coronavirus 2; SEM, standard error of the mean.

## 4 DISCUSSION

The Innova LFD is widely used in the United Kingdom, with government data showing a high level of clinical specificity.^13^ However, data from multiple testing sites showed a sensitivity of only 37.4%–57.4% compared to RT‐PCR.^13^ Our data is in agreement with this; using 52 positive patient samples, we found a sensitivity of 46.2%. It should be noted that the samples tested in this study were diluted, which may affect this value. However, our study was carefully designed to ensure the same volume of sample was tested on PCR and LFD. It should also be noted that this study tested frozen samples, which may affect sensitivity. Samples were handled in the same way for both PCR and LFD testing, with the majority frozen in Igepal, which stabilizes proteins.

An argument supporting the lack of sensitivity of LFDs is that they detect “live” viruses as inferred by cell culture experiments.^7^, ^14^ The theory is that they should identify patients who are contagious. While a proportion of our patients may be carrying residual RNA, it is likely that some patients with a low viral load are at an early stage of infection. Data published by Public Health England show that samples with Ct values of 32.5 can contain live virus (1.2 pore‐forming unit [PFU]/ml), while detection by LFD is very poor above Ct 28.5 (40 PFU/ml).^15^ In a separate study, it was shown that viable SARS‐CoV‐2 virus can be cultured from samples with Ct values >35.^16^ A recent UK study, utilizing infection of close contacts as a proxy for contagiousness, proposes a strong correlation between Ct value and likely transmission.^17^ While it is probable that someone with a high viral load (low Ct) is more contagious, transmission cannot be ruled out in people with a low viral load. Data from the United States clearly shows that Ct values do not predict transmissibility—mean Ct values for spreaders and nonspreaders were almost identical with the transmission still observed when the index case had a Ct > 36.^18^

The Innova LFD failed to detect SARS‐CoV‐2 in samples with viral loads of 3 and 1.5 million copies/ml. This was also noted during large‐scale community testing where LFD sensitivity was only 67% in samples with a Ct value <25.^19^ Furthermore, the studies cited above used cell cultures to test for the live virus and infer infectivity of a patient.^7^, ^14^ This data should be interpreted with caution; data from Switzerland showed that outbreaks occurred where the first three cases had viral loads of <100,000 copies/ml.^20^ Our study focussed on the Innova LFD as this is supported in the United Kingdom. Alternative LFDs may have a superior performance.

Ct values only provide a guide on viral load and there is no consensus on a Ct value, which corresponds to infectiousness.^7^, ^21^ We used digital PCR to correlate Ct values with viral loads. Previous studies examined samples with an unspecified viral load.^13^ Where viral load was calculated, standard calibrants were used, a method that is less accurate than digital PCR,^11^, ^15^, ^17^, ^22^ Ct values of 25.5 were considered to equate to 100,000 copies per/ml in one study^15^ with 24.4 equalling 10,000 copies/ml in another.^17^ Using digital PCR, Ct values of 25.5/24.4 were equivalent to 17/40 million copies/ml, respectively. Our data matches other publications where digital PCR was used to calculate SARS‐CoV‐2 viral load.^22^

It should be noted that 50% of the positive LFD tests produced a very faint positive result (see Table 1) across all replicates. This may not be scored as a positive result by an untrained individual. The validity of self‐administered tests and thus sample quality remains a concern and previous data showed that LFD sensitivity was reduced from 79.2% to 57.5% when tests were performed by members of the public compared to a trained individual.^15^ The justification for using LFD devices is that they are cheap and can produce a result in around 30 min while bypassing the requirements for rigorous laboratory standards. Our streamlined laboratory PCR procedure is able to produce results in as little as 90 min from sample receipt.

Using highly accurate digital PCR^11^ to calibrate viral load was a major strength of this study and something which was absent from similar diagnostic accuracy studies. A further strength of this study was the access to positive samples of a known viral load— this allowed the selection of samples with a wide range of viral loads allowing for a robust comparison of techniques. Comparison with a rapid RT‐PCR method is very relevant as this method can be comfortably scaled up for mass testing. One limitation of this study was the low sample number—this was overcome by selecting samples with a range of viral loads in triplicate. A further limitation was the use of frozen samples, which may affect sensitivity. However, the use of frozen samples has been reported in comparable studies.^23^, ^24^

In conclusion, the Innova LFD was found to be up to 10,000 times less sensitive than a rapid RT‐PCR; therefore, a negative result should be interpreted with caution. In the United States, the Food and Drug Administration has issued a Class I recall for the Innova LFD, citing concerns over performance.^25^ Despite this, the Innova LFD remains approved for use in several countries, including France, Germany, Italy, and Turkey, and is widely used in the United Kingdom.

## AUTHOR CONTRIBUTIONS

**Andrew Taylor:** Conceptualization; data curation; formal analysis; investigation; methodology; project administration; writing–original draft; writing–review and editing. **Ronan Calvez:** Conceptualization; data curation; formal analysis; investigation; methodology; project administration; writing–review and editing. **Mark Atkins:** Conceptualization; resources; supervision; writing–review and editing. **Colin G. Fink:** Conceptualization; resources; supervision; writing–review and editing.

## TRANSPARENCY STATEMENT

The lead author Andrew Taylor affirms that this manuscript is an honest, accurate, and transparent account of the study being reported; that no important aspects of the study have been omitted; and that any discrepancies from the study as planned (and, if relevant, registered) have been explained.

## Supporting information

Supporting information.

## Data Availability

The data that support the findings of this study are available from the corresponding author upon reasonable request.
