# Supplementary material for: Comparing lateral flow testing with a rapid RT‐PCR method for SARS‐CoV‐2 detection in the United Kingdom—A retrospective diagnostic accuracy study
Source: Health Sci Rep. 2022 Sep 14;5(5):e811. doi: 10.1002/hsr2.811 (PMC9475223; doi:10.1002/hsr2.811)
Supplement: Supplementary file 1 — Supporting information. [file HSR2-5-0-s001.docx]

**Table S1**: Details of patient samples used for this study. Correlation values refer to the Pearson correlation calculated using Microsoft Excel.

| **Gender** |  | Correlation (r) with Ct value | Correlation (r) with LFD score |
| --- | --- | --- | --- |
| Male | 32 |  |  |
| Female | 30 |  |  |
|  |  | -0.035 | 0.0025 |
| **Age** |  |  |  |
| Mean | 38.8 |  |  |
| Median | 34.5 |  |  |
| Range | 4-92 |  |  |
|  |  | -0.10 | 0.18 |
| **Original CT of positives** |  |  |  |
| Mean | 30.2 |  |  |
| Median | 30.7 |  |  |
| Range | 18.9 - 36.5 |  |  |
